# Teaching common cognitive biases and associated errors in veterinary neurologic diagnosis using clinical vignettes: a narrative review

**DOI:** 10.3389/fvets.2026.1940970

**Published:** 2026-09-18

**Authors:** Rod Bagley

**Affiliations:** Department of Veterinary Clinical Sciences, College of Veterinary Medicine, Iowa State University, Ames, IA, United States

**Keywords:** clinical evaluation, cognitive bias, cognitive error, neurologic diagnosis, teaching

## Abstract

Clinical evaluation and management of veterinary patients is a human endeavor and therefore dependent on the capacity and limitations of human cognition in forming clinical opinions of patient health and decisions relative to patient care. Consequently, numerous cognitive biases are pervasive in the clinical evaluation of animal patients. These biases include, but are not limited to, representativeness bias, confirmation bias, availability bias, selection bias, and overconfidence bias. While these mental constructs can support positive patient outcomes through rational thinking, recognition of common clinical patterns, and intuitive experiences, in other instances these cognitive biases can contribute to cognitive error and medical decision-making mistakes. The astute clinician is cognizant of when cognitive bias becomes a cognitive limitation to compensate for, and ideally avoid, the potential negative consequences of these decision-making strategies. Most importantly, these mental phenomena can and should be formally taught to veterinary students as a component of learning the clinical method, so developing clinicians become familiar with these important influences on clinical thinking as they cultivate their clinical acumen. This narrative review explores teaching common clinical judgment biases using contextual clinical case vignettes which illustrate the operational dynamics associated with these biases during clinical assessments. By identifying and actively debriefing on how clinical thinking paradigms influence case situational dynamics, aspiring clinicians will improve their clinical judgment benefiting clinical science, patient care, and ultimately, patient safety.

## Introduction

Diagnosis and management of animal neurological disease is contingent upon a clinician’s skill in observation, patient examination, pathophysiological localization, problem identification, and interpretation of diagnostic testing results. The clinician’s thinking and reasoning during clinical evaluation is predicated on contextual knowledge, empirical experience, focused attention, sensory skill, and processes which are often difficult to articulate such as “intuition.” All these cognitive processing elements are aspects of “clinical reasoning” or simply, how and what the clinician “thinks” during the diagnostic evaluation (1–9).

Throughout all aspects of the clinical evaluation process there are numerous human cognitive processes that influence clinical decision-making (10–17). Each clinical encounter utilizes a combination of conscious and subconscious (“instinctive” or “intuitive”) decision-making, commonly referred to in psychology as “System 1” and “System 2” thinking (18). The latter is based on acquiring knowledge and information that is consciously accessible and utilized when stimulated. The former processes, i.e., “intuition” is a complicated mix of previous nervous system stimuli and resultant reflexive actions that affect the decision-making processes. This intuition subsequently influences conscious decision-making. This intuitive process is sometimes equated with “experience” and is developed with repeated neural activation from clinical patients through senses such as seeing, hearing, smelling, and touching. As the process of how personal intuition is acquired is often difficult for individuals to articulate, it is equally difficult to teach students and early career clinicians how to develop these clinical skills.

Some of these cognitive processes are viewed as positive (such as problem identification, integrating complex data patterns, and problem solving) and result in positive patient outcomes (such as making an accurate diagnosis). Others are known cognitive limitations that, if not recognized and managed, can result in misdiagnosis, inappropriate diagnostic and treatment decisions, and ultimately, negative patient outcomes (19–28). Given the potential negative consequences to the patient, the skilled clinician is aware of, and metacognitive regarding, these cognitive limitations during their patient evaluations.

Preferences for making certain decisions within certain situations known as cognitive biases are pervasive in medical settings and replete in clinical experiences (29–60). While often used interchangeably, the terms cognitive bias, cognitive error, and cognitive illusion represent distinct but interrelated psychological phenomena that affect clinical decision-making (10, 13, 15, 19–22). Experts who study decision-making processes vary somewhat in their individual definitions of specific cognitive biases with a scientific consensus taxonomy of each of these cognitive phenomena is still developing (61). Additionally, within the complexities of human decision-making thinking there is overlap and blending of individual biases which makes explicit separation of one cognitive event from another difficult. These are reasons why a single cognitive bias is often conflated with other cognitive biases when retrospectively debriefing clinical evaluation scenarios. The existence in allied professions including human medicine as well as observations in the veterinary profession establish cognitive bias and cognitive error as realities in veterinary clinical practice (60, 62–70).

The mental strategies resulting in cognitive bias are systematic, predictable patterns of deviation from an established objective reality and are commonly the outcome of using heuristics (10–17). Heuristics are natural mental constructs or cognitive “shortcuts” developed by the human brain to efficiently assess data and rapidly make decisions from that data (71–81). These constructs bias decisions based on often limited but relevant information adapted to a specific decision-making environment (75). Heuristics are influenced by the context and conditions in which they are performed and may be used with both positive and negative outcomes (78). Within medical decision-making, well adapted heuristics to the environment can be used effectively to make appropriate clinical decisions when there is limited information and with limited time for decision-making such as in emergency situations (75). In other environments and contexts, however, heuristics can result in less-than-optimal decision-making (78). Factors including stress, cognitive load, illness, degree of wakefulness, emotional activation, mental distraction, and time pressure contribute to use of heuristics in decision-making, all of which are common dynamics within medical practice (82–90).

This review will focus on teaching components of cognitive processing including default preferences, biases, and decision-making preferences that occur during clinical examinations and diagnostic interpretation of patients with neurologic disease using clinical vignettes as contextual examples of how these biases manifest in a clinical encounter. Given that logical clinician decision-making is a part of veterinary skill development, teaching veterinary medical students the consequential cognitive biases and errors as a component of their clinical skill development should be an explicit element of veterinary clinical competency training relative to clinical decision-making. Teaching cognitive biases using clinical scenario review is one potential debiasing learning strategy (32, 91–96). While there are myriad cognitive biases, some are more frequently encountered in clinical veterinary neurological practice and are emphasized in this review for student education (Table 1). These same cognitive dynamics, however, are prevalent throughout all veterinary medical practice and should be considered ubiquitous in every veterinary clinical diagnostic evaluation.

**Table 1:** Types of cognitive bias common in clinical practice.

Name	Description
Representativeness bias	When clinicians estimate the likelihood of a disease based on how closely a patient’s signs align with a “stereotype” or prototype of that disease expression
Confirmation bias	The tendency to search for, focus on, interpret, favor, and recall information in a way that confirms or supports one’s prior assumptions and beliefs
Hindsight bias	The tendency, upon learning the outcome of a decision, to subsequently overestimate one’s ability to have foreseen that particular outcome from this retrospective perspective
Availability bias	A distortion that arises from the impact of information which is most readily available (from memory), rather than that which is representative of the current state
False cause or correlation error	Failure to determine the appropriate cause for a specific outcome
Anchoring	Initial information is more influential on decision-making compared to information acquired subsequently in the discovery process
Framing	When an individual’s choice from a set of options is influenced by how the information is worded or presented as compared to the actual information presented
Appeal to authority	Abdicating responsibility for a decision to an authority
Intervention (action) bias	Judging harm caused by doing something as less than harm from no action
Omission (inaction) bias	Judging harm caused by not doing something as less than harm from an action
Outcome bias	Judging the quality of the decision by the outcome of that decision
Selection bias	The natural bias of only evaluating evidence or data that was selected
Overconfidence bias	The tendency for an individual to overestimate their abilities

Using case vignettes as contextual examples affords the student the opportunity to reflect on when these biases can occur and how these biases may result in cognitive errors through the associated learning outcomes of each scenario (Table 2). The case scenarios provided are abridged from actual clinical cases as compared to purely theoretical scenarios to demonstrate how these biases and errors often manifest in “real world” clinical evaluation (60). During the review students are asked to first identify the potential biases in each case scenario. In some situations, the bias is obvious and independent. In other instances, multiple biases may be occurring simultaneously making clear distinctions difficult as to which bias is most influential in any single patient encounter. Many of these are “blends” of bias with elements of different cognitive biases occurring in parallel (61). Next, the students explain how each of the biases influence the medical decisions made. Finally, the students articulate strategies of how to mitigate or avoid these errors using alternate decision-making paradigms. These clinical scenarios demonstrate clinical thinking errors that are, unfortunately, observed consistently in debriefing patient encounters when teaching clinical neurologic evaluation and should not be viewed as isolated occurrences.

**Table 2:** Learning outcomes of case scenario review.

Learning outcomes
Identify any cognitive biases occurring in this scenario
Explain how each of the biases identified influence the medical decisions made
Articulate strategies to avoid associated cognitive errors based on the cognitive bias

For each scenario more than one cognitive bias and or cognitive processing errors may be operational, however, one of the potential cognitive biases will be accentuated for additional discussion and emphasis. When there are multiple potential cognitive biases within each scenario, the teacher/facilitator can choose which ones to emphasize within each clinical teaching scenario and debrief as appropriate based on the learning outcomes for that educational session.

### Case scenario A

A five-year-old male castrated Dachshund presents with paraparesis resulting from a lesion localized to the thoracic (T)3 to lumbar (L)3 spinal cord (Figure 1). As intervertebral disc disease is generally known as common in this breed, and can result paraparesis, a clinician decides that the clinical signs are “representative” of intervertebral disc disease as a cause of this patient’s dysfunction (97, 98). That is, the clinical features were consistent with a known stereotype for an animal with intervertebral disc disease. In case scenario A, following additional diagnostic information utilizing magnetic resonance (MR) imaging, this dog is found to have a traumatic spinal injury with mild subluxation at T8-9 intervertebral space cause of dysfunction (Figure 2). A presumptive diagnosis of “intervertebral disc disease” is thus proven inaccurate given this additional diagnostic information.

**Figure 1:**
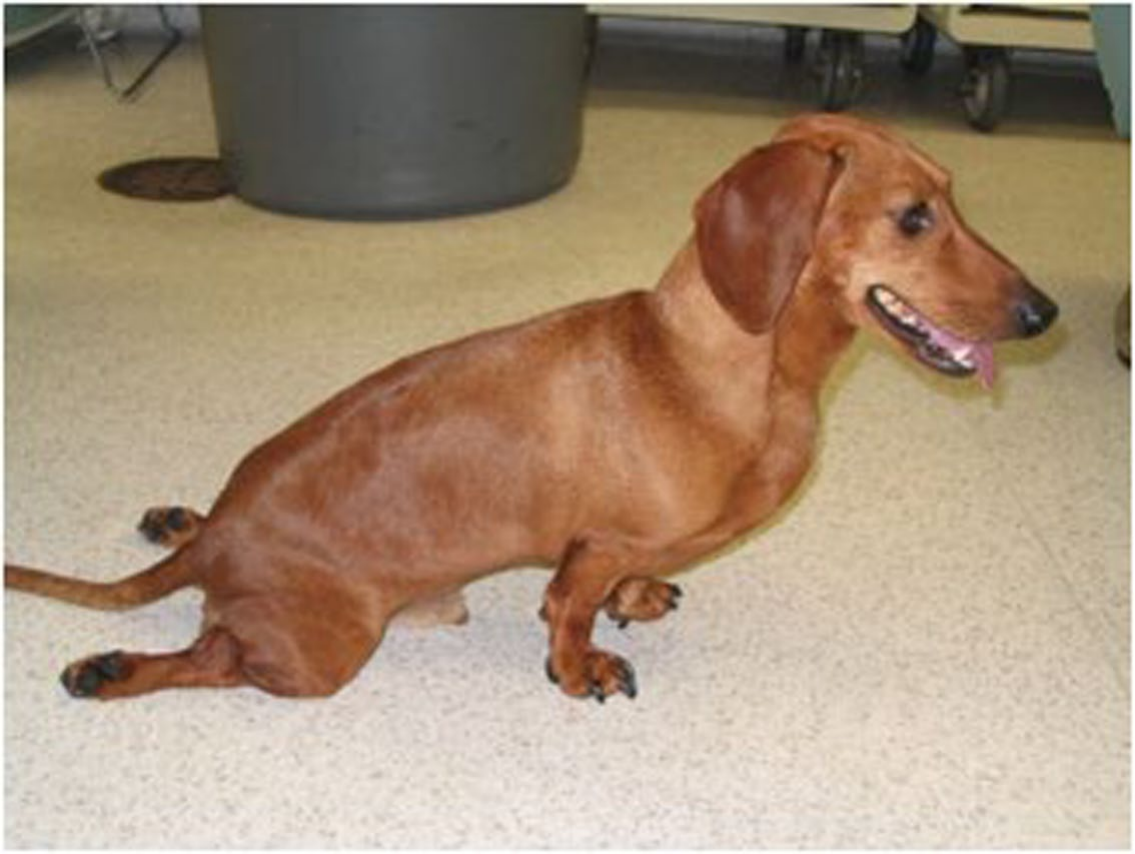
A Dachshund that is unable to stand and move is often presumptively diagnosed with intervertebral disc disease based on the association of this breed and this disease.

**Figure 2:**
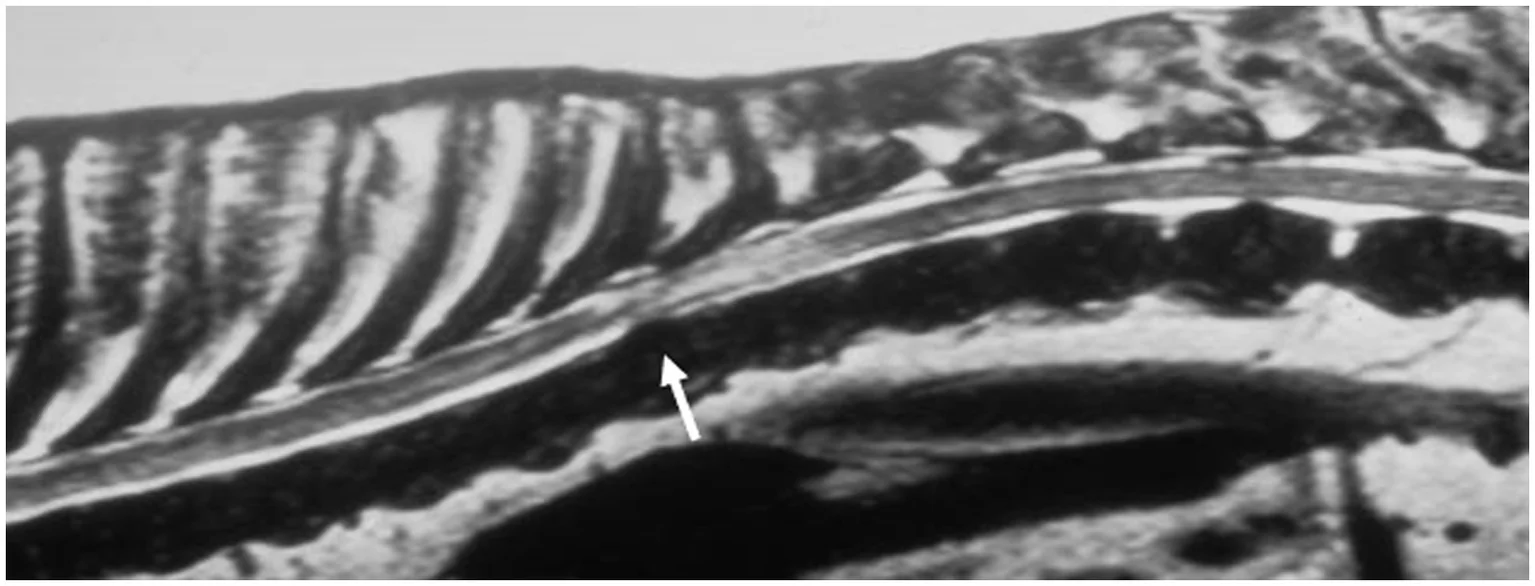
Sagittal T2-weighted MR image from the patient demonstrating a spinal alignment abnormality and associated spinal cord injury associated with a mild vertebral subluxation at the T8-9 intervertebral space (arrow).

### Case scenario B

A seven-year-old male castrated German Shepherd Dog presents with 4 months of progressive ambulatory paraparesis. The neuroanatomical localization is a non-painful T2-L3 myelopathy. An initial diagnosis of “degenerative myelopathy” is rendered (99). Subsequently, an MR imaging study of the thoracolumbar spinal region demonstrates three adjacent intervertebral disc protrusions at T11-12, T13-L1, and L1-L2 (Figure 3). Decompressive surgery via a hemilaminectomy is performed and the protruded intervertebral disc material is removed (Figure 3). Over the ensuing 4 weeks the dog’s clinical signs improved with minimal residual paresis. Twelve weeks following surgery the dog is clinically normal.

**Figure 3:**
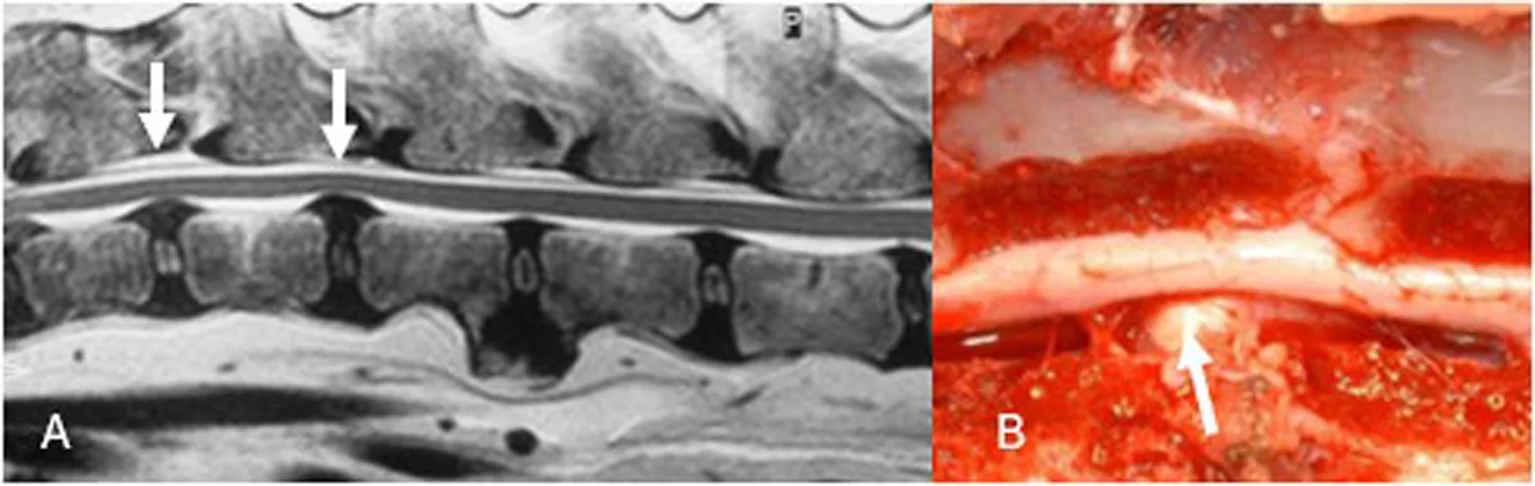
Sagittal T2-weighted MR image. **(A)** Demonstrating multiple intervertebral disc protrusions (arrows). Intraoperative view. **(B)** Following hemilaminectomy demonstrating the appearance of one of the intervertebral disc protrusions (arrow).

### Case scenario C

A four-year-old female spayed Labrador retriever dog is presented following an acute onset of inability to stand in the pelvic limbs while fetching a ball. At the time of the acute onset of the clinical signs the dog vocalized. Upon examination, the dog is paraplegic with normal spinal reflexes, normal mentation, and normal cranial nerve function. Neuroanatomical diagnosis is a T3 to L3 spinal cord abnormally. A presumptive diagnosis of a “fibrocartilaginous emboli” (FCE) is made.

Survey radiographs of the thoracolumbar spine are normal. Subsequent MR imaging demonstrates a traumatic intervertebral disc extrusion at L2- L3 intervertebral space (Figure 4).

**Figure 4:**
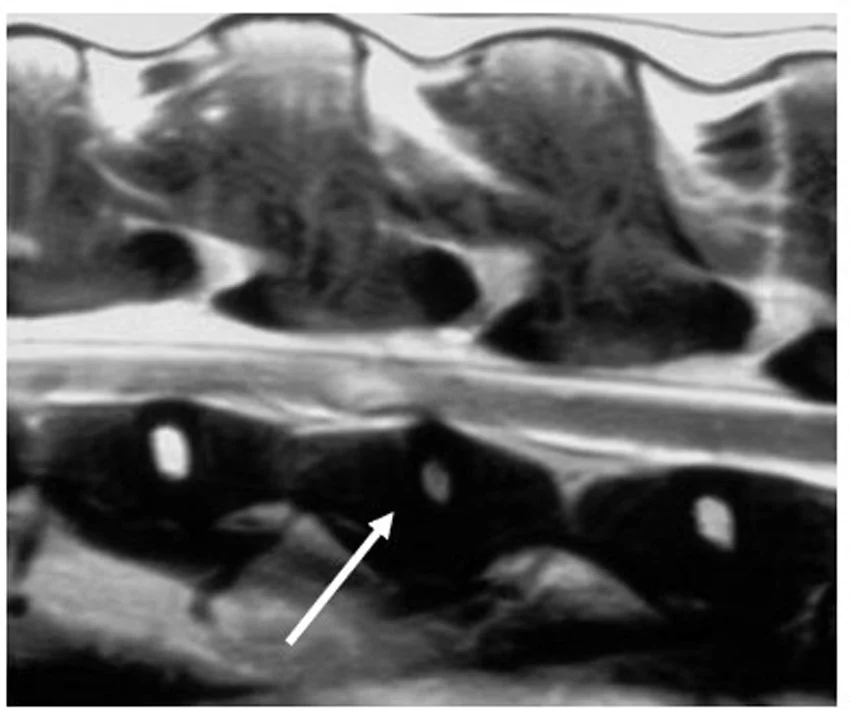
Sagittal T2-weighted MR image demonstrating a traumatic intervertebral disc extrusion (arrow).

### Debrief discussion of representativeness bias

Representativeness bias occurs when something is judged to be associated with a particular group based on how closely that something aligns with an existing stereotype or prototype (11, 13, 15, 19–22, 100, 101). In medical evaluation, a representativeness bias can occur when patient’s clinical characteristics are judged to be associated with a specific disease based on how closely that patient’s clinical characteristics align with an existing stereotype or prototype of patients with that disease. The initial clinical diagnosis, therefore, is a form of representativeness bias utilizing a representativeness heuristic wherein the clinician estimates the probability of disease diagnosis based on how closely the patient’s clinical features match a historically recorded disease presentation stereotype. With appropriate knowledge, experiences, memories, and observational skills, this intuitive assumption is likely to be more accurate than chance which is why this process is an accepted expectation of clinical decision-making within a probabilistic reasoning framework.

If, however, the patient’s clinical characteristics are more nuanced or there is greater individual diversity than those used to establish the stereotype, placing the patient in a general category of patients with similar but generic clinical features can be inaccurate resulting in a representativeness error. A representativeness error occurs when the clinical diagnosis, based on stereotypic disease features, is not the actual disease this is affecting the patient. As illustrated by these cases, given the limited data points (three is this instance—breed, non-specific clinical signs, and a spinal cord localization), this patient could have numerous different specific disease processes resulting in these same clinical signs and in this neuroanatomical location. Attributing the cause of the clinical signs to a specific disease process when more than one disease can result in the same clinical signs is a frequent clinical judgement error (19–28). This error is more likely to occur when the information considered is limited and generalizations are made about a patient’s disease based on this limited information. Representativeness errors may also occur when there is a diverse expression of the disease phenotype.

As all diseases are expressed in an individual patient based on that individual’s unique physiology and pathophysiology, this creates a significant conundrum for clinicians as even the fundamentally same disease process may be manifested in any individual patient differently from the typical disease stereotype (Figure 5). There is a tendency in retrospective evaluations of disease expression to group individual patients into disease categories based on having the same disease etiology, such as intervertebral disc disease. Co-morbidities, however, are often under articulated, under contemplated, or not even considered in assessing the expression of the target disease in an individual patient. Obvious factors influencing overall disease expression include age, environment, nutrition, and genetics. Equally, abnormalities in non-neurologic body systems such as degrees of abnormality in liver, heart, kidney function, blood pressure, and any other pathophysiologic derangement within the body can alter the expression of neurological disease within that individual. All these factors make generalized assessments of disease expression difficult to predict and may contribute to a representativeness error.

**Figure 5:**
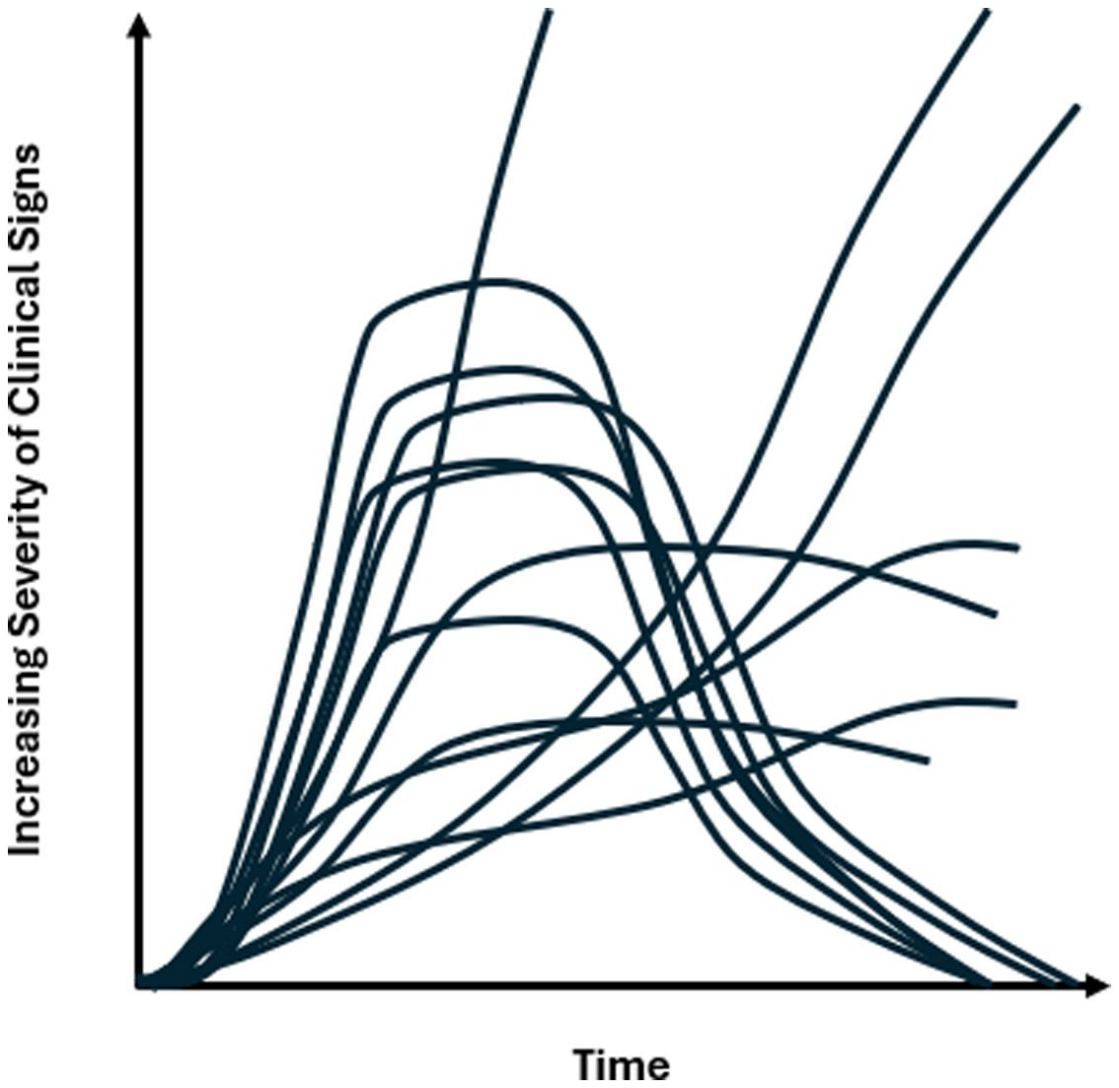
Sign-time relationship from multiple patients with the same disease and diverse disease patterns.

In situations similar to case scenario B, when an initial clinical diagnosis of “degenerative myelopathy” is made based on clinical signs, and more advanced diagnostic tests such as MR imaging are subsequently performed, many patients presumptively diagnosed with “degenerative myelopathy” are found to have other spinal conditions; the most common being chronic intervertebral disc protrusion. In this specific scenario chronic intervertebral disc protrusions result in similar clinical features to those of degenerative myelopathy and should be considered in a patient with these similar clinical features. Case scenario C illustrates a very common situation in contemporary veterinary neurology where there is confusion in diagnosis between patients with FCE and patients with acute extrusion of nucleus pulposus into the spinal canal (the latter is sometimes referred to as “acute, non-compressive nucleus pulposus extrusion” or “ANNPE”) (102–107).

These and similar case scenarios can be used to help students understand that there are numerous disease processes with similar clinical signs and to avoid correlation errors based on representativeness. These case scenarios emphasize the importance of not stopping further diagnostic inquiry and search satisfying when the clinician is confident of a disease process in a patient because that patient fits a disease stereotype. Representativeness is often aligned with other cognitive biases such as availability bias, confirmation bias, and anchoring which could be equally operational in similar cases as discussed independently as learning opportunities in these case examples.

### Case scenario D

A four-year-old male castrated dachshund is presented with difficulty walking and moving in a forward direction. When the dog walks, the dog assumes a kyphotic posture in the thoracolumbar spine and the dog’s inter-step distance in the pelvic limbs is shorter than normal (Figure 6). The dog can walk for a few steps but then sits down and does not move further. The dog has paw placing (proprioceptive) deficits with weight-bearing but has normal paw placement proprioception when the examiner supports the dog’s body weight. Additionally, the dog has normal spinal reflexes, normal mentation, and normal cranial nerve function except for an inability to close the palpebral fissure when the medial canthus of the eye lid is touched. The initial examining clinician had seen five cases of Dachshund diagnosed with “intervertebral disc disease” within the last 2 months and these cases were memorable to the clinician during the evaluation of this patient. Based on the information that this is a dachshund with pelvic limb dysfunction and the memory of other similar cases of dachshunds with intervertebral disc disease, the clinician makes a presumptive diagnosis of “intervertebral disc disease” as the cause of this dog’s clinical signs. With reflective assessment, however, the clinical signs in this dog are more indicative of a peripheral nerve, neuromuscular junction, or muscle disorder. Ultimately, during a subsequent reassessment, the dog is diagnosed with myasthenia gravis.

**Figure 6:**
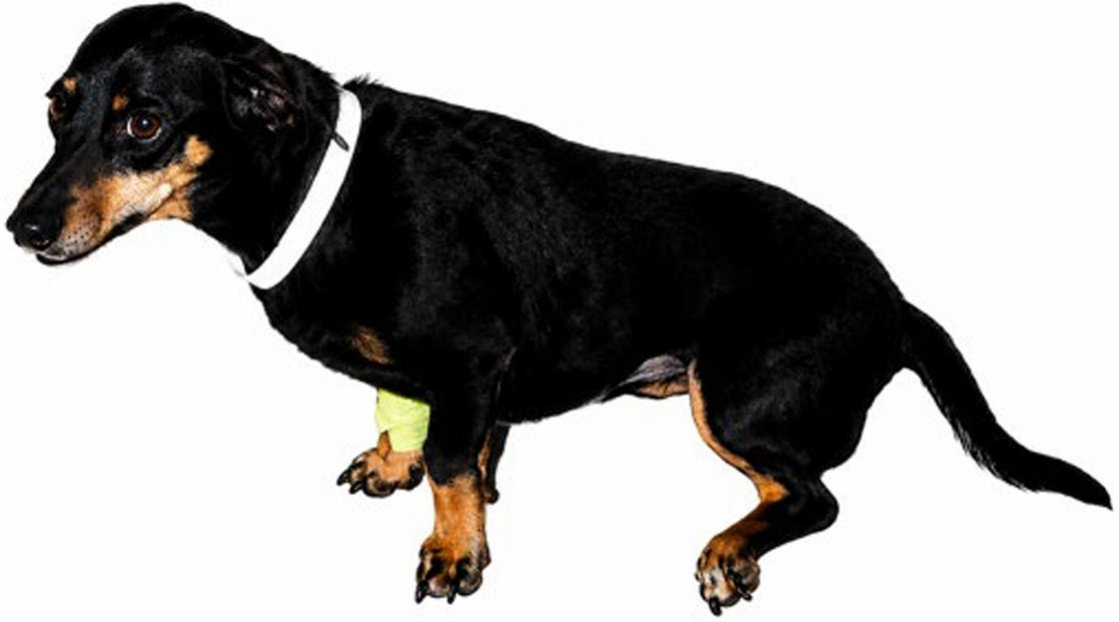
Appearance of the dog with short inter-step distances and inability to walk a short distance before stopping.

### Debrief discussion of availability bias

Availability bias is a mental distortion that results from the influence of information which is most readily available (that is, from memory), rather than that which is indicative of the current state (11, 13, 19–22, 108, 109). It is a natural human tendency to remember certain events/occurrences more vividly and these events are subsequently perceived as occurring more commonly or being more likely. These situations are, simply stated, more “memorable.”

“Memory” as a cognitive capacity influences all reflective thinking and an element of many cognitive errors. “Experience” is, in essence, the process of accumulating memories. The individual patient encounter becomes a component of the clinician’s mental “database” stored as a memory. The frequency and recency of acquired information are important factors in what is remembered (75). Errors in “misremembering” form a foundational basis of numerous judgment errors including representativeness, availability, confirmation, hindsight, and outcome bias (58, 110, 111).

An availability error occurs when the clinician diagnoses a disease that they remember or is immediately memorable, but the patient subsequently has a different disease process that the clinician did not consider. Another nuance of availability is not just recently memorable cases, but what disease processes the clinician can recall at the time of the clinical evaluation. When diseases are not considered as in the situation presented here because the clinician does not mentally recognize the disease as a possibility this may be that the clinician does not know that a disease (in this scenario, myasthenia gravis) is a possibility in this breed of dog or that the clinician did not correlate the clinical signs of shorten inter-step distances, normal proprioception with examiner support, and the decreased palpebral reflex as being consistent with a different disease process as compared to a spinal cord disease. These latter situations would be errors of basic knowledge, inexperience with the clinical expression of an individual disease (in this case, myasthenia gravis), or failure to recognize the clinical nuances of a neuromuscular cause of the movement abnormality. Errors that result in the actual disease not being considered or identified are often referred to as the disease being “missed.” This terminology implies that these diseases were not considered by the clinician for a variety of reasons.

This scenario helps students to understand that clinical knowledge must both be generally established and be “available” under the circumstances encountered. In clinical evaluation, the number of different diseases on a differential diagnosis list is a tangible example of how many disease processes are “available” to the clinician’s mind at the time of patient assessment. Assessing the ability of a student to formulate a list of diseases would be an outcome measure of what is “available” to them in a specific patient encounter.

Finally, in some instances of clinical practice a disease process is generally not known to exist or known to exist but assumed to be infrequent. Therefore, this disease is generally not “available” for consideration. One historical example of a disease not being “available” to the clinician is syringomyelia (112–114). Syringomyelia has, in fact, been known to occur for years, however, was considered “rare” and therefore, not considered as a significant cause of neurologic dysfunction in animals (Figure 7). Given the now established ubiquitous nature of syringomyelia this disease is found frequently in clinical patients, this disease is now “available” to the clinician as a differential diagnosis for spinal cord disease presentations. These types of situations help students understand that there are new diseases that are yet to be identified or are uncommon that still require consideration.

**Figure 7:**
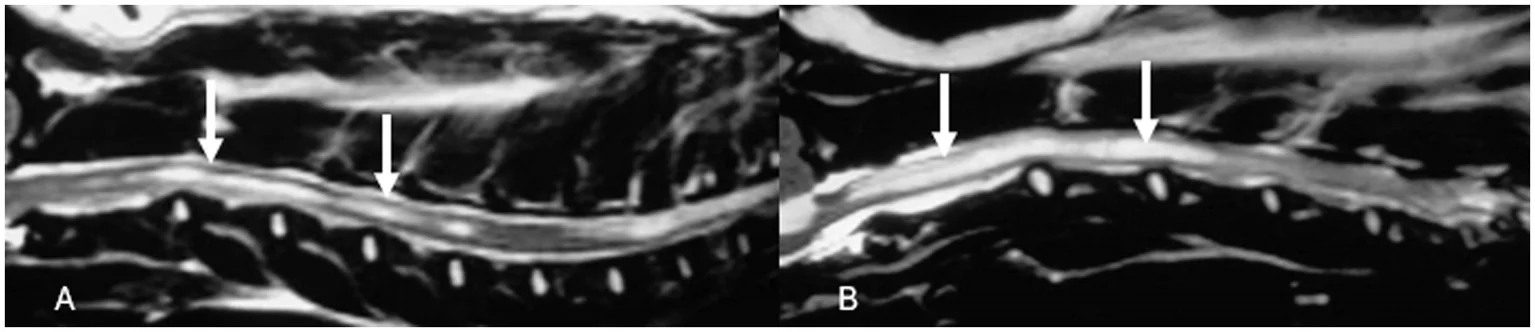
Sagittal T2-weighted MR images **(A,B)** from two dogs demonstrating syringomyelia (arrows).

### Case scenario E

A three-year-old male castrated French Bulldog, a breed known to have intervertebral disc disease, presents with paraparesis (115). The dog is presumptively diagnosed with “intervertebral disc disease” based on clinical information such as appropriate breed, clinical signs and, familiarity of base rates of disease in this breed.

Based on these clinical decisions, the clinician elects to perform computed tomography (CT) of the thoracolumbar spine which demonstrates multiple vertebral abnormalities of the thoracic vertebra without obvious spinal cord compression (Figure 8). The CT findings now “confirm” another common abnormality in the spine of this breed (115). Given the equally common vertebral abnormalities in this breed, the clinician decides that these vertebral abnormalities are the cause of the dog’s clinical signs and stops searching for other diseases which can result in similar clinical signs.

**Figure 8:**
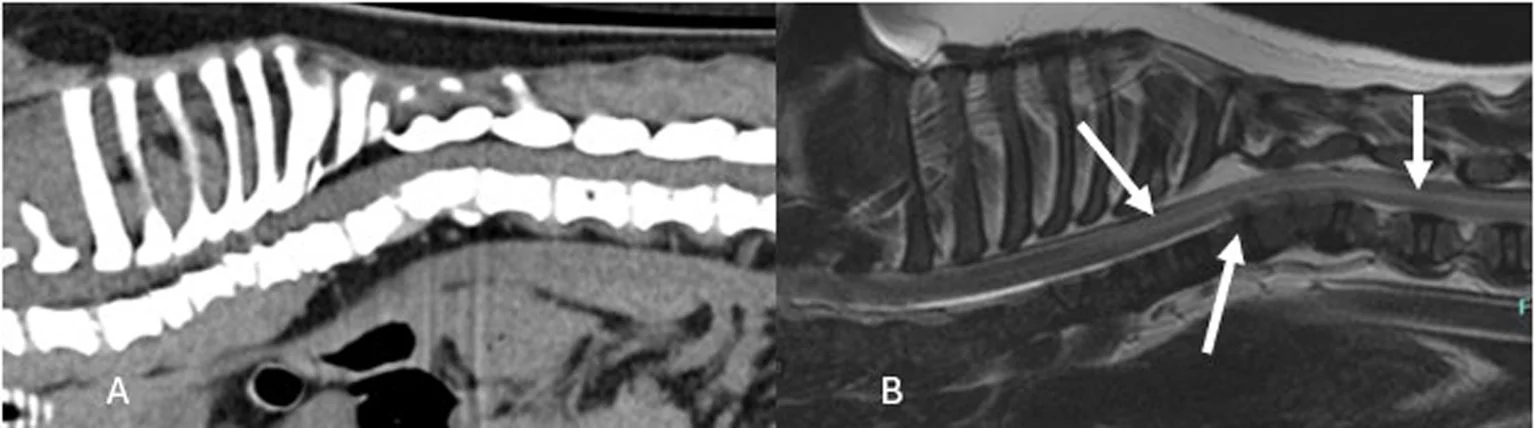
Sagittal CT **(A)** and T2-weighted MR image **(B)** from the same level. The CT image demonstrates multiple vertebral abnormalities. The MR image demonstrates spinal cord compression and likely syringomyelia (arrows) that was not apparent on the CT image.

### Debrief discussion of confirmation bias

Confirmation bias is the tendency to search for, focus on, interpret, favor, or recall information in a way that confirms or supports one’s prior assumptions and beliefs (11, 13, 15, 19–22, 42, 43, 116). In essence, we work to confirm what we already “believe” or, at least, our default mental process is to accept our own conclusions before attempting to disprove them (11, 13, 15, 19–22). “Believing” is a foundational aspect of clinical practice given the inherent uncertainties at multiple stages of the clinical evaluation process (117, 118). Beliefs in diagnosis are often articulated with the terms such as “clinical” or “presumptive.” Recognizing a belief as a hypothesis of disease which is unproven permits the clinician to be receptive to new information that develops throughout patient evaluation and subsequently alter any belief as the clinical diagnostic process is refined. If the clinician stops searching for additional clinical abnormalities based on confirmation of one spinal abnormality, potentially additional conditions will not be identified, and an incomplete or inaccurate diagnosis will be made.

This bias commonly overlaps with overconfidence (that is, we have already formulated the conclusion so this conclusion must be accurate) and availability bias (that is, we are focusing on the things we know and discounting the possibility of things we cannot or choose not to mentally consider). Confirmation and similar retrospective assessments such as hindsight and outcome bias are augmented when clinicians learn the outcome of a patient’s condition which is why clinical research methods often include “blinding” to interventions to avoid associating any intervention with outcome.

If each decision “confirms” what the clinician is thinking relative to an etiologic diagnosis, the clinician may not engage in thinking about alternate possibilities and consider different pathophysiologic processes that result in the same clinical signs. In this way, susceptibility to a confirmation bias tends to limit additional information gathering resulting in premature closure of clinical diagnostic decision-making with failure to consider alternate diagnoses.

A confirmation bias can result in a confirmation error when a patient does not have the disease that was initially clinically formulated. This confirmation error thus results in an initial inaccurate clinical hypothesis and may subsequently delay accurate assessment of a patient’s abnormality or prevent reassessment through reevaluation of the initial conclusion. These diversions of attention can result in the potential for a patient’s disease to persist or worsen prior to appropriate interventions or to be simply misdiagnosed. Confirmation bias also promotes a tendency to continue existing treatments rather than reevaluate the treatment decisions previously rendered.

One contemporary example is the trend in some hospitals to exclusively utilize CT imaging in animals with spinal disease to confirm a presumptive diagnosis of intervertebral disc disease or a vertebral abnormality (119–129). Computed tomography imaging does not, however, adequately demonstrate the numerous other potential disease processes and pathophysiological sequalae that may be present in any individual patient even when a conspicuous disease processes such as a calcified intervertebral disc extrusion or vertebral abnormalities are identified. In this case scenario, an additional diagnostic evaluation using magnetic resonance (MR) imaging was performed that demonstrated spinal cord compression at T8-9 and intraspinal cord hyperintensity consistent with a pathologic process increasing the water content of the spinal cord such as edema, syringomyelia, inflammation (Figure 8). As many of the studies comparing CT to MR evaluations are based on either (1) a presumptive diagnosis of a suspected disease such as intervertebral disc disease and (2) a “definitive” diagnosis “confirmed” by surgical observation, these situations demonstrate additional nuances of confirmation bias.

Another clinical dynamic is that many neurologic diseases are “recoverable” and therefore the clinician is never challenged to reconsider their clinical decisions throughout the recovery process. Finally, clients cannot consistently pursue definitive diagnostic testing, making disease confirmation not possible in all cases. In reflecting on confirmation bias the students are reminded to continue their diagnostic inquiry beyond what is the apparent conclusion to the case diagnosis.

### Case scenario F

A four-year-old male castrated mixed breed dog is found unable to use the pelvic limbs however with voluntary movements in the limbs intact (i.e., “non-ambulatory” paraparesis). The neuroanatomical localization is a T3-L3 spinal cord disease. Following formulation of a different diagnosis list for spinal cord diseases, an MR imaging study is performed of the thoracolumbar spine. An intervertebral disc extrusion is present at the L1-L2 intervertebral disc space (Figure 9).

**Figure 9:**
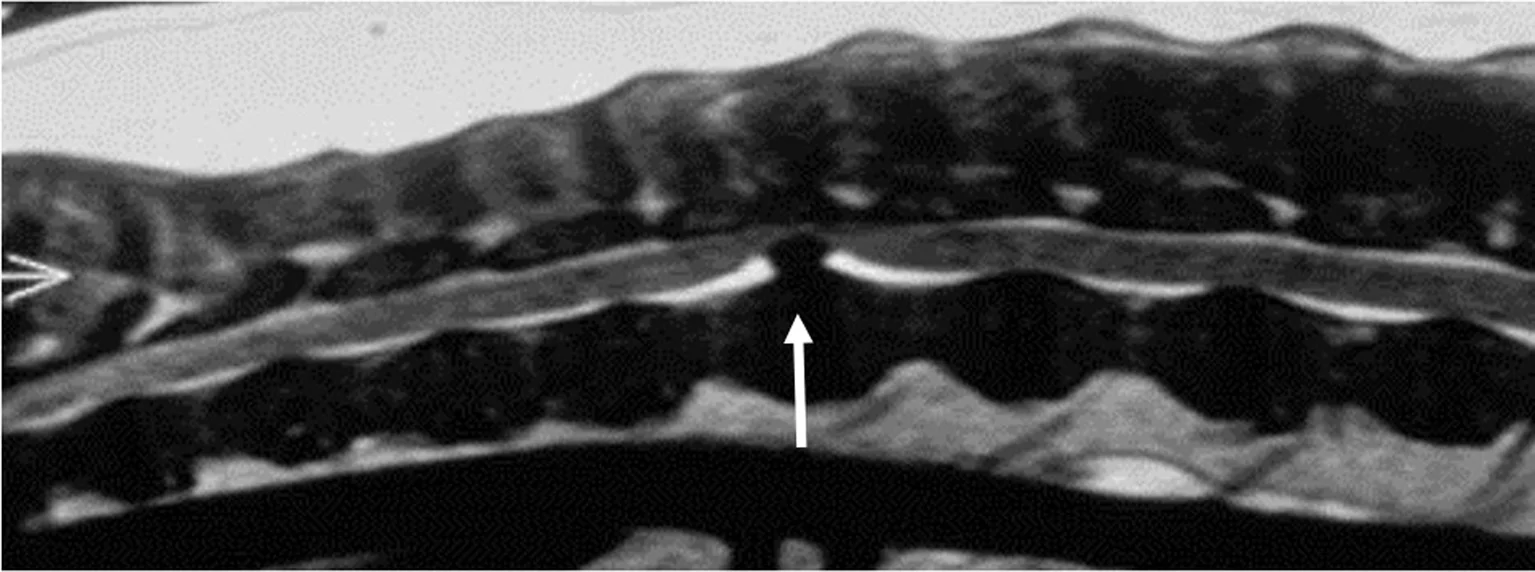
Sagittal T2-weighted MR image from the dog in Case Scenario F. The MR image demonstrates spinal cord compression from an intervertebral disc extrusion (arrow).

A decision is made to surgically decompress the spinal cord and a hemilaminectomy is performed at that site. The dog eventually regards the ability to stand and walk over the next 12 weeks. At the 12 week follow up examination the conclusion is made that the surgery was the reason that the dog recovered the ability to walk.

### Debrief discussion of hindsight and outcome bias

Hindsight bias is the tendency, upon learning the outcome of a decision, to subsequently overestimate one’s ability to have foreseen that particular outcome prospectively (11, 13, 15, 19–22, 130–137). When the outcome that happens is the one that was “expected,” then it is more likely to conclude that the initial clinical assessment was, in fact, “correct” independent of whether this same outcome could be merely a result of “chance” or a mental “guess.” Hindsight bias intersects with overconfidence (supporting the belief that clinical thinking is cogent and sound), confirmation bias, and other causal assessments by providing the illusion of precognition and preemptive accuracy in a prospectively unknown diagnosis.

An outcome bias has some similarities to hindsight bias and confirmation bias in assessing the decision-making process based on the outcome of the decision (11, 13, 15, 19–22, 136, 137). All these biases are more likely to occur when animals improve or recover (such as recovering independent limb movement) and are judged to do so because of a medical action without equal consideration that these animals would improve or recover spontaneously. An outcome bias can occur in situations where patients do well (positive outcome) and equally in those situations where the patient does poorly (negative outcome). This bias prospectively influences decisions made on subsequent patients with similar clinical diagnoses. This bias can be associated with any patient outcome and the decisions that predicted that outcome including treatment decisions such as surgery, acupuncture, rehabilitation, chiropractic manipulation, laser, and numerous other non-surgical treatment applications. Similar to hindsight bias, an outcome bias can result in an inaccurate assessment that the clinical decision-making process was sound when the outcome was, in fact, independent of these clinical decisions. One aspect of mitigating these retrospective evaluative biases is educating students of the patient’s outcome from the natural course of the disease independent of any intervention by the clinician.

One way to teach students the natural disease expression without intervention is what is known as the “sign-time” relationship (138–142). If a disease process has a known sign-time relationship, the clinician who evaluates a patient at any time point in the temporal relationship of the clinical signs will be able to accurately predict the future course of the clinical signs (Figure 10). The temporal course of the disease is usually taught with the basic facts associated with a disease process. When reflecting on group scientific information such as a randomized clinical trial this is essentially the “control” population manifesting the unadulterated course of the disease pathophysiology. Understanding the natural course of the disease in the individual patient unaltered by any specific medical intervention allows for a determination of any improvement in the disease course in a target population receiving a medical intervention.

**Figure 10:**
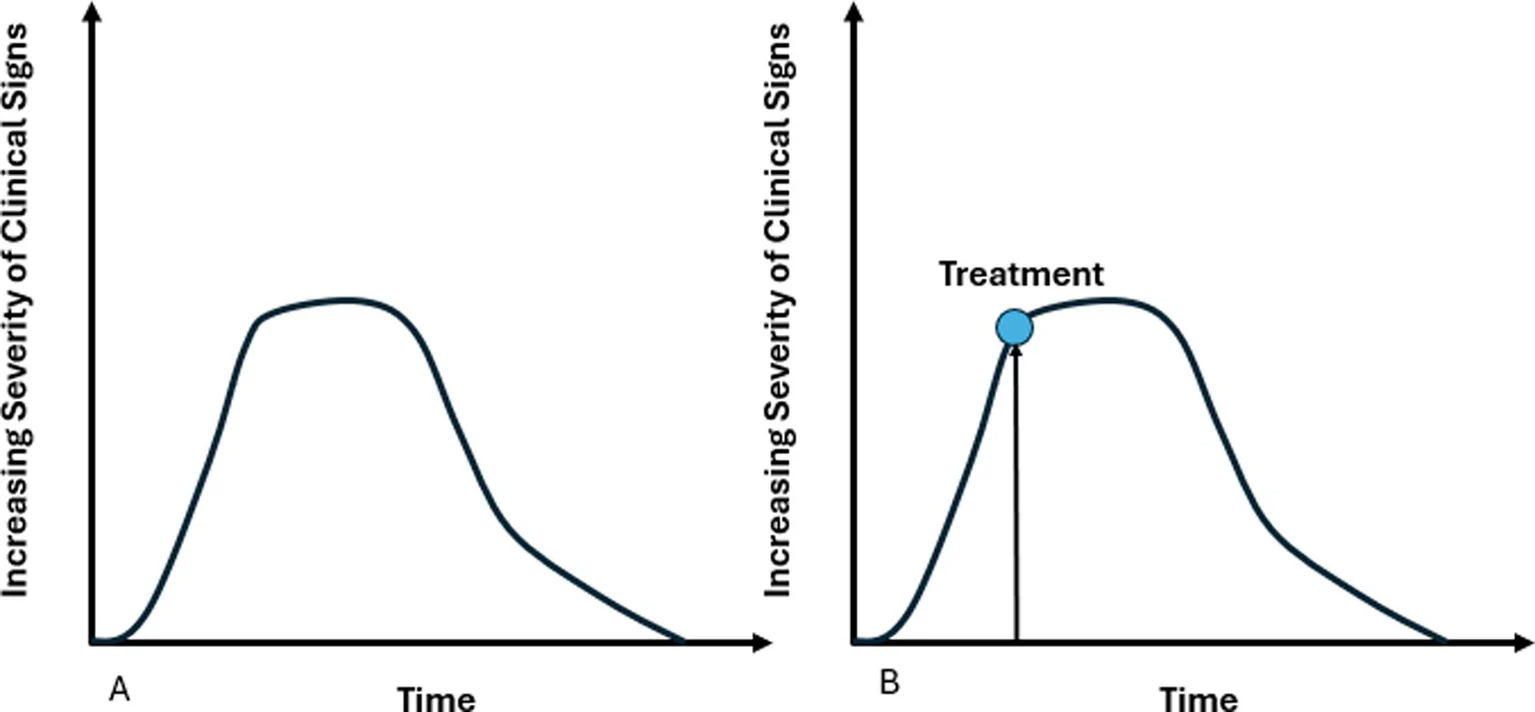
Sign-time relationship demonstrates **(A)** the natural course of a recoverable disease and **(B)** that same disease pattern following a treatment intervention. With this information there is no treatment effect as the disease naturally improves over time independent of any intervention. The treatment in this instance does not affect the natural course of the disease.

Within this context, the association of clinical outcomes directly resulting from a medical intervention are notorious situations for misinterpretation of cause and effect (15, 143–146). It is a default bias to believe that an intervention results in a positive effect if the clinical signs improve within a temporal relationship following the intervention. From a clinical neurology context, it is known that a certain percentage of dogs with clinical signs of intervertebral disc extrusions will spontaneously recover walking (that is, without a specific treatment) at a future time and commonly within 4 to 16 weeks following the onset of initial clinical signs (147–161). When students become familiar with the natural disease course, however, they are better able to compare clinical outcomes from interventions such as surgery, acupuncture, or chiropractic manipulation as significant or not.

For example, if it is known that a disease pattern will have resolving clinical signs at a future specific time, any treatment response within this same time should be viewed as potentially insignificant compared to the natural healing process (Figure 10). We can conclude, then, that a single patient improving based on a medical intervention is not conclusive evidence of a treatment effect and that “response to therapy” is not diagnostic evidence of a definitive disease process in that patient. This is also the case with chronic diseases without clinical improvement where the progression of the disease process is not affected by a medical intervention (162). The greater the number of patients evaluated the more likely the diverse spectrum of clinical disease expression will be codified. This is an opportunity to teach the importance of assessing “sample size” is a salient feature of interpretating clinical data.

When students are familiar with this information, they are better able to assess the effectiveness of any treatment intervention for patients with spinal disease. Equally, they can offer a more accurate prognosis for the owner based on a spectrum of clinical interventions.

### Case scenario G

A three-year-old female spayed cat is presented following being hit by a car. Immediately following the vehicular impact, the cat is unable to stand in the pelvic limbs. The cat is paraplegic with normal spinal reflexes, normal mentation, and normal cranial nerve function. Neuroanatomical diagnosis is a T3 to L3 spinal cord abnormality. Survey radiographs of the thoracolumbar spine demonstrate a fracture of the caudal aspect of the body of the T12 vertebrae (Figure 11). No definitive treatment is performed, and over the next 3 months the cat returns to being able to independently stand and walk relatively normally.

**Figure 11:**
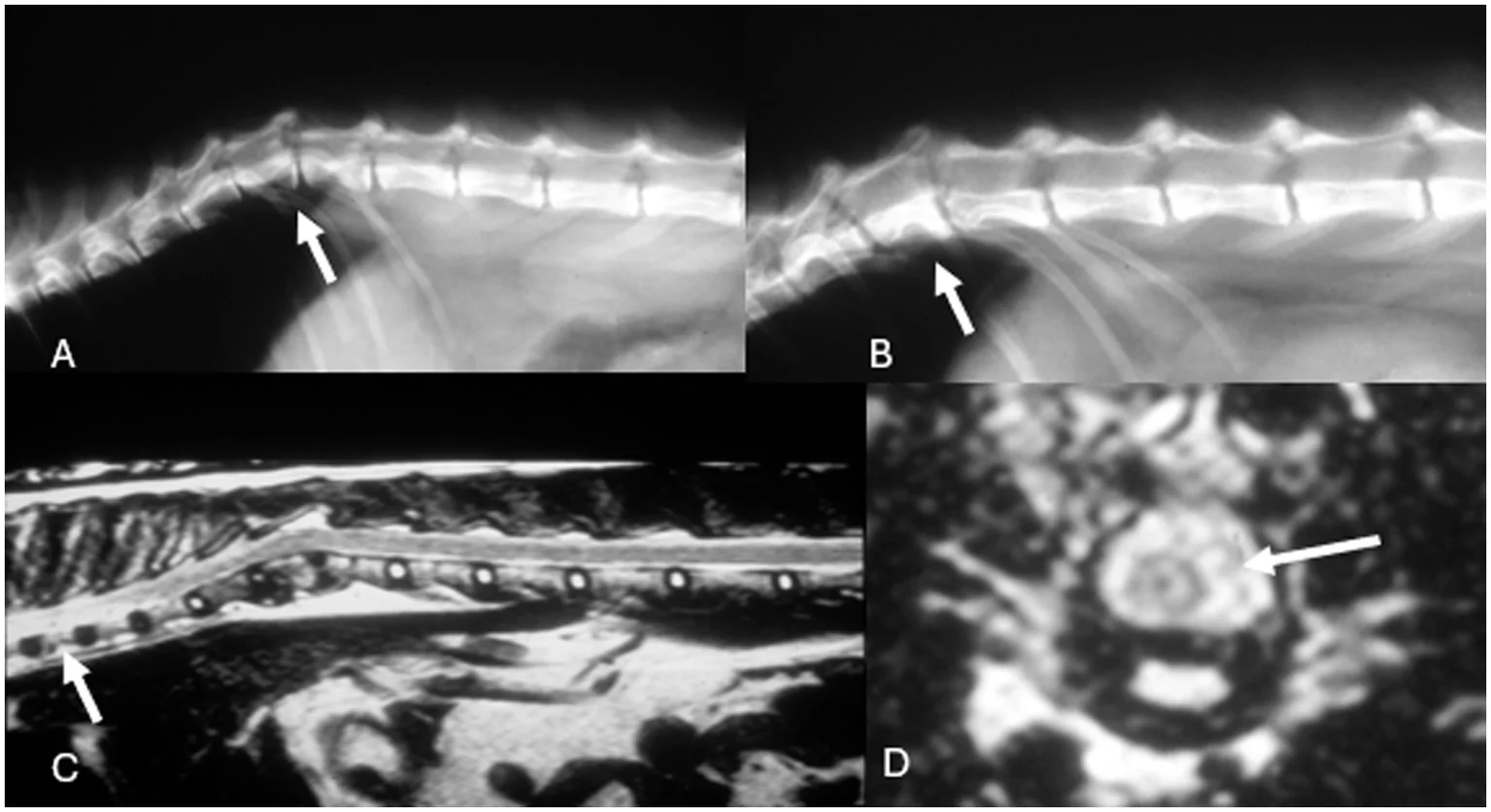
Lateral survey radiograph of this cat from the first presentation **(A,B)** demonstrating a caudal vertebral body fracture at T12 (arrows). Sagittal **(C,D)** T2-weighted MR image at the second presentation demonstrating no spinal compression at the previous injury site, however an extradural mass in the mid to caudal thoracic region (arrows). Histologic diagnosis of the mass was lymphosarcoma.

Eight months following the cat’s recovery the cat again becomes acutely paraplegic with normal spinal reflexes, normal mentation, and normal cranial nerve function. Neuroanatomical diagnosis is a T3 to L3 spinal cord abnormality. Survey radiographs of the thoracolumbar spine demonstrate malalignment of the spine at the previous fracture of the body of the T12 vertebrae (Figure 11). At this visit, however, MR imaging is performed and demonstrates that the spinal cord is not abnormal (other than orientation) at the fracture site, however, there is diffuse extradural spinal cord compression in the cranial thoracic spinal cord region cranial to the previous fracture site (Figure 11). Hemilaminectomy is performed at this site, and an extradural spine cord soft tissue mass lesion is identified. Subsequent biopsy of this mass demonstrates lymphosarcoma.

### Case example H

A ten-year-old male castrated German Shepherd Dog presents for an acute onset of paraplegia. When collecting historical information, the owner states that the dog jumped from the bed of a pick-up truck to the ground 2 days prior to the onset of clinical signs. The patient is assessed based on the previous information of a potential traumatic injury to the spine during the jump. Following a diagnostic evaluation the dog is found to have a spinal tumor (osteosarcoma) of the T2 vertebrae resulting in spinal cord compression (Figure 12). Surgical removal of this tumor results in short term clinical improvement in the dog’s clinical signs. The tumor, then, was the likely cause of the clinical signs at this presentation, and the historical “traumatic incident” is likely serendipitous.

**Figure 12:**
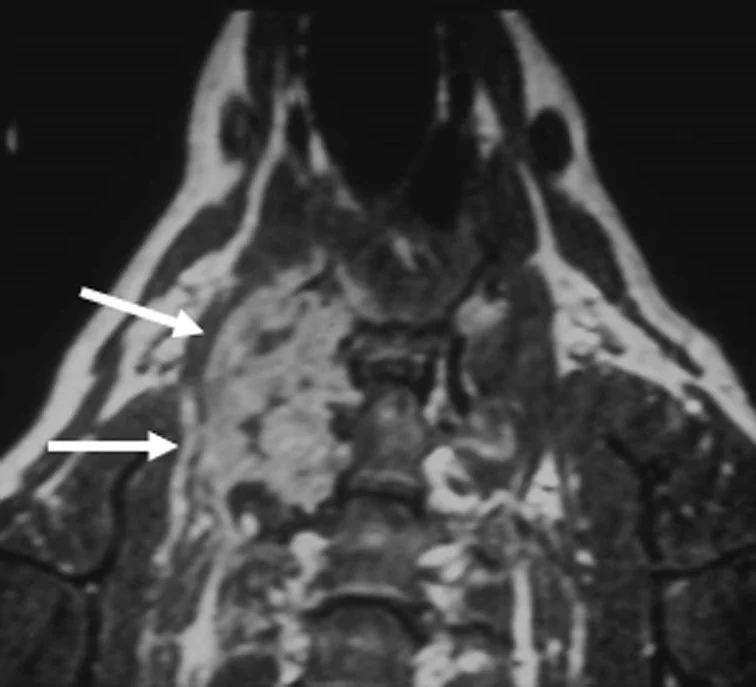
Dorsal T2-weighted MR image demonstrating a neoplastic mass (arrows) involving multiple vertebral in the caudal cervical and cranial thoracic region of the dog.

### Case scenario I

A two-year-old female spayed border collie dog is presented following being hit by a car. Immediately following the vehicular impact, the dog is unable to stand and assumes a lateral recumbent position. The dog is tetraparetic with normal spinal reflexes, normal mentation, and normal cranial nerve function. Neuroanatomical diagnosis is a cervical (C) 1 to C5 spinal cord abnormality. Survey radiographs of the cervical spine demonstrate a fracture of the body of the C5 vertebrae (Figure 13). Subsequent MR imaging demonstrates that the spinal cord is not damaged at the fracture site, however, there is evidence of a traumatic intervertebral disc extrusion at C3-4 intervertebral space (Figure 13). The clinical signs associated with either a spinal fracture or an intervertebral disc extrusion overlap, therefore, if a diagnosis of a cervical fracture as the cause of the signs was made solely from the cervical radiographs, the actual cause of the dog’s clinical disability (that is, the traumatic intervertebral disc extrusion) would have been missed relative to the more apparent vertebral fracture.

**Figure 13:**
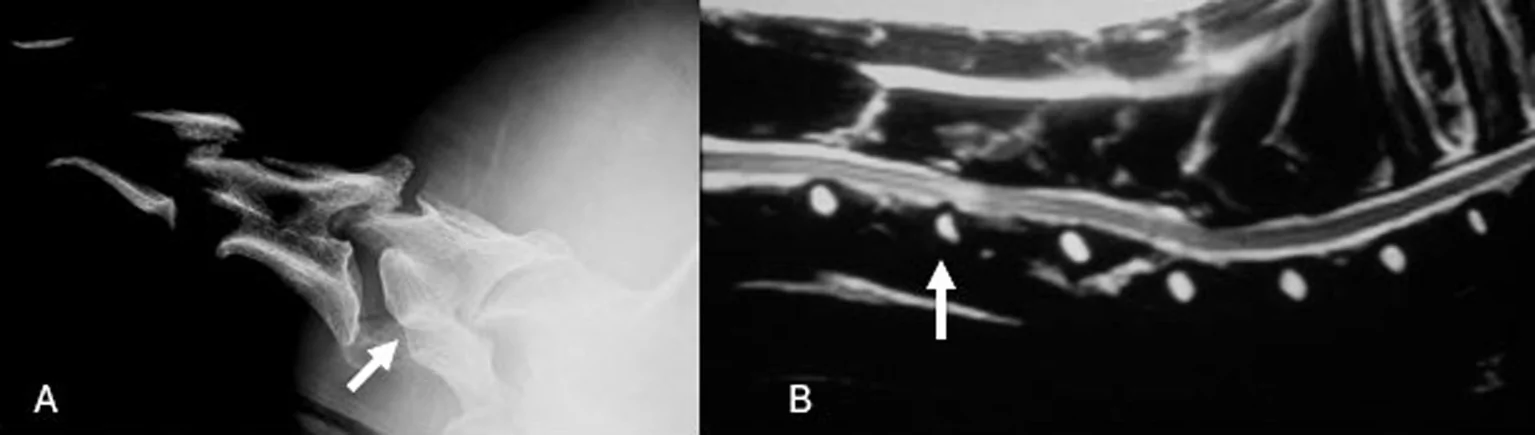
Lateral survey radiograph **(A)** and sagittal T2-weighted MR image **(B)** from the dog in case scenario I with a C5 fracture involving the vertebral body (arrow). On the MR image there is no spinal cord abnormality at C5 associated with the fracture, however, there is traumatic intervertebral disc extrusion at the C3-4 intervertebral space (arrow).

### Debrief discussion of anchoring and framing biases

Anchoring is a cognitive bias wherein information gathered initially is more influential on decision-making as compared to information acquired subsequently in the discovery process (11, 13, 15, 19–22, 163–169). This can occur any time new information is presented, including during the clinical examination process or when receiving diagnostic testing reports. Anchoring bias may overlap with confirmation bias when a clinician has initially made what they believe is a reasonably formulated diagnosis and remains “anchored” to this initial diagnosis. As with many of the other biases, a tendency to become “anchored” can prevent the exploration of multiple different scenarios for a single patient situation.

Framing or the framing effect is the cognitive bias wherein an individual’s choice from a set of options is influenced by how the information is worded or presented as compared to the actual information itself (12, 14, 170–174). As all information collected is essentially “framed” in a certain context, it is obvious how these dynamic elements influence medical decision-making. Diagnostic tests are another form of framing which provides a distinct “view” of the patient from the nuanced perspective of that test. Framing, like other cognitive biases, places mental boundaries that limit possibility thinking.

In case scenario G the cat had identical signs and disease courses at two independent clinical presentations, however, with potentially different pathological causes. The clinical signs associated with either a spinal fracture or a spinal cord tumor overlap, therefore, if a diagnosis of a reinjury to the spine was made at the second presentation solely from the historically presented (“framed”) information and the radiographs, the actual cause (that is, the spinal cord neoplastic disease) would have been missed.

Historical information also serves to anchor the clinician’s thought process toward any previous diagnosis and a failure to realize that each clinical presentation can be the result of a different disease process. In this case scenario, the second visit is framed in the context of information from the initial presentation and diagnosis.

In case scenarios H and I, the observed traumatic incidences anchored and or framed the initial thought processing toward traumatic injuries as the cause of each dog’s clinical signs. In case scenario I there was additional evidence from the initial radiographs demonstrating a vertebral fracture that that abnormality was the cause of the dog’s clinical signs. If the diagnostic search was stopped following the radiographs, the actual cause of the dog’s clinical signs would have been missed. As with many of the previously mentioned cognitive biases, the ability to consider more than one possibility as to the cause of the clinical signs helps to counter these types of assumption errors.

### Case scenario J

A six-year-old female spayed mixed breed dog presents with paraparesis resulting from a lesion localized to the T3 to L3 spinal cord. Prior to consenting additional diagnostic testing, the owner asks for the prognosis for their dog to recover the ability to walk. The clinician searches on-line for information regarding recovery percentages of animals with intervertebral disc disease and reviews three manuscripts (156, 157, 175). These manuscripts provide recovery percentages for dogs with intervertebral disc extrusions and surgical treatments. Based on this information, the clinician informs the owner that the prognosis of their dog walking in the future is 80%.

### Debrief discussion of selection bias

A selection bias naturally occurs when individual cases are chosen for inclusion in any retrospective or prospective analysis for either a research study or for inclusion in an individual clinician’s mental case “database” (176–184). This bias is a form of sampling bias where data included for analysis is non-random. There are multiple types of selection bias (184). With selection error, the data (information) “selected” for evaluation is not representative of the target population and provides a biased perspective of a disease or patient population (11, 13, 15, 19–22, 176–184). Selection bias and subsequent error may be overt or covert. When overt, clinicians only include their observations or analysis from a subset of any population. A form of selection bias, attrition bias occurs when only patients that have positive outcomes or that complete an assessment are included for analysis creating a bias in making conclusions only from this set of patients and ignoring those that did not have positive outcomes or complete the observation period. These latter patients are often described as “lost to follow up.” If all patients within a patient population are not included for analysis this also results in a reporting bias when sharing that information with colleagues (180).

As another nuance of selection bias, a clinician evaluates 12 cases of a similar disease in their patient population. This collection of patients becomes the individual clinician’s selected group representing that disease process either for inclusion in a research analysis or their own mental database for that disease. If, however, the number of patients in the world with a similar condition number in the hundreds of thousands, this individual clinician’s experiences are a small sample of this target population. It is often underappreciated that any clinician, regardless of the spectrum of their individual clinical experiences, will not evaluate *every* clinical presentation of *every* clinical case in a population. Therefore, any clinician’s experiences are, by definition, “selected.” This bias shares some common elements with other aligned biases such as framing, anchoring, confirmation and availability bias.

### Case scenario K

A four-year-old female spayed mixed breed dog is found unable to use the pelvic limbs however with voluntary movements in the limbs intact (i.e., “non-ambulatory” paraparesis). The neuroanatomical localization is a T3-L3 spinal cord disease. Following formulation of a different diagnosis list for spinal cord diseases, an MR imaging study is performed of the thoracolumbar spine. An intervertebral disc extrusion is present at the T13-L1 intervertebral disc space. The clinician informs the client that the only option to improve the clinical signs is surgical decompression of the spinal cord.

### Debrief discussion of action (commission/interventional) and inaction (omission) bias

In clinical activities there can be both a bias toward either doing something or doing nothing. With an action bias an individual would have a preference for action compared to inaction. In medicine, an interventional bias is the bias on the part of physicians to intervene, when not intervening would be a reasonable alternative (18–22, 185). For example, a bias toward performing surgery compared to allowing the disease course to naturally occur. An action bias may also occur as a defensive strategy if the risks of not acting have negative personal consequences such as litigation resulting in overdiagnosis and overtreatment (75). This creates a bias toward actively treating a disease. In essence, this is the mental idea that “doing something is better than doing nothing.” This is not to imply that any medical intervention is inappropriate under certain circumstances but rather that when the scientific basis for deciding on the most appropriate treatment is not available there is a default toward action (treating). This bias is perpetuated by owner expectations or the perception of clinicians as to what owners expect in treatment actions. Interventional bias can occur in early career clinicians who may not have the experiences of the natural course of a disease independent of a medical intervention and, to avoid being criticized for inaction, treat. A commission bias can result in a clinical error by having patients be treated with therapies that are either not beneficial or, in the worst case, detrimental to their recovery.

An inaction or omission bias is judging harm caused by not doing something as less than harm from an action (11, 13, 15, 19–22, 31, 186). This bias may result in the opposite of an interventional bias wherein inaction is chosen even when inaction is associated with higher risks or worse outcomes (186). Either of these biases may be unique to the individual clinician and therefore, not uniformly predictable in any given clinical decision.

### Case scenario L

An eleven-year-old male castrated German Shepherd dog is presented for “back pain. The neuroanatomical localization is a T3-L3 spinal cord disease. Following formulation of a different diagnosis list for spinal cord diseases, lateral survey radiograph is performed of the thoracolumbar spine (Figure 14). These radiographs were submitted to an on-line diagnostic imaging interpretation service for review by a board-certified radiologist. The radiology report states, “*The vertebrae are normal*.” A second opinion identifies the obvious spondylosis deformans present (Figure 14).

**Figure 14:**
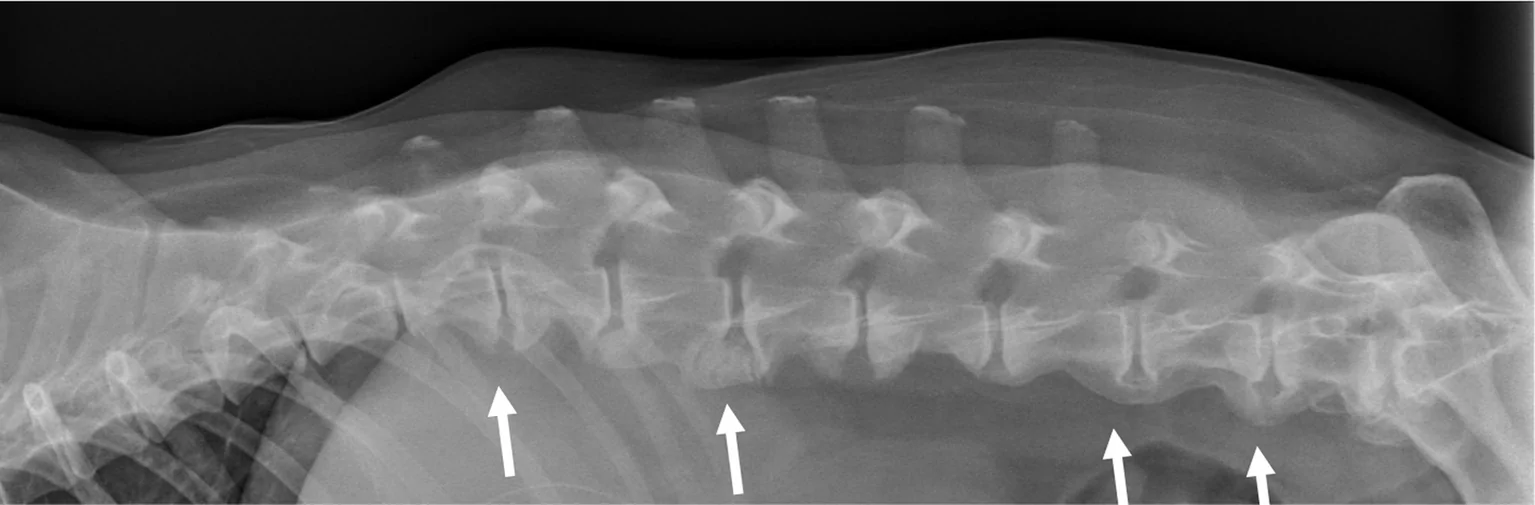
Lateral survey radiograph of the dog of case scenario L. This radiograph demonstrates spondylosis deformans of the thoracolumbar vertebrae (arrows).

### Debrief discussion of appeal to authority

Appeal to authority is fundamentally abdicating the responsibility for clinical decision-making to another who is recognized as an authority in the subject (16). This example includes the modern practice of referral to a specialist or securing a specialist’s opinion. When a clinician lacks advanced knowledge in an aspect of clinical practice, it is standard of practice to engage other expert opinions in that process. In the contemporary era, the surge of teleradiology is another obvious example of appealing to an authority for a clinical decision.

Appealing to an authority in making a clinical decision is valid if the individual rendering the opinion is, in fact, an “authority.” In contemporary clinical practice this is usually assumed based on evidence of advanced training and certification by examination. It is equally valid that specialists are susceptible to the same types of cognitive biases under discussion and, therefore, can be fallible in certain circumstances. The most effective method to avoid inappropriately “appealing to authority” is to gain the requisite knowledge and skills in current best practices or standards of care.

### Overconfidence bias within clinical practice

Overconfidence bias is the tendency for a person to overestimate their abilities (11, 13, 15, 19–22, 187–191). This phenomenon is often stated as the most common cognitive bias of human beings as we naturally tend to believe what we believe, think, and experience. This is also a person thinking that they are “better-than-average.” The Dunning-Kruger effect is one paradigm to examine this relative to the development of “expert performance” and/or “competency” related to self-confidence (192). In this relationship, the initial slope of the Dunning-Kruger effect may be the most damaging of the cognitive illusions as overconfidence precludes metacognition and leads to “ignorant bliss.”

Given that clinical judgment is a potential high-risk activity requiring the need for one to be “confident” to “move forward” in juxtaposition with the need to be accurate and/or “competent,” there is a tendency toward “overconfidence” as compared to “under confidence.” This cognitive bias also epitomizes the dichotomy between “confident” (the feeling that “I can”) compared to competency (“I did”). From a clinical “teaching” perspective, this has relevance to using direct as compared to indirect outcomes assessments in assessing clinical performance. The errors associated with being overconfident relative to competence result in actions that are inappropriate for the clinician’s skill level.

### Other considerations in clinical cognitive bias for discussion

As there are a plethora of potential biases that can influence clinical outcomes, it is important that the aspiring clinicians familiarize themselves with all of these to recognize when and how these biases may be influencing their clinical decision-making. Additionally, there may be novel biases and errors that will develop with changes in how clinical diagnosis is made in the future. As an example, automation bias is an overreliance on automated systems for decision-making (80). As diagnostic algorithms become generated by computer-based data assessments it will be important to determine how these dynamics influence future clinical decision-making (192–200). Equally, how will clinical decision-making be made when clinical thinking is abdicated to a non-human source of information? These and other nuanced biases will need to be considered as diagnostic processes alter the future of clinical diagnosis and treatment decision-making.

### Addressing cognitive bias in teaching clinical evaluation

To mitigate a potential cognitive bias resulting in a cognitive error is a significant professional challenge for the experienced as well as the aspiring clinician. For students, these cognitive biases should be explicitly discussed when teaching clinical decision-making and become an acquired clinical skill to increase diagnostic accuracy and ultimately, benefit the patient. There are numerous options that have been utilized for teaching debiasing (49, 201–237).

Debiasing techniques are structured strategies designed to interrupt unconscious, emotional, or intuitive decision-making processes (49, 201–237). The most common debiasing interventions include education (awareness) of cognitive bias in clinical evaluation, following a checklist, and practicing developing skills with clinical vignettes that demonstrate cognitive bias and the influence on diagnostic error (49, 201–237). Education is a common first practice to address these errors proactively. Some strategies also employ reflective reasoning exercises, “thinking aloud” processes, or explicit instructions such as writing differential diagnosis lists. Unfortunately, many of the current debiasing interventions have surprisingly limited to non-existent positive effects on diagnostic accuracy or decreasing medical error at present (49, 201–237). This fact suggests that additional and potentially different strategies aimed at addressing cognitive bias in medical practice are still needed.

To reduce representativeness and availability bias, formulating a robust list of potential diagnoses in any individual patient to prevent cognitive fixation on a limited spectrum of disease processes is obligatory. As students are taught facts related to individual diseases, they should concurrently be challenged to create extensive and appropriate differential diagnoses lists from a problem list of patient abnormalities. Teaching students the importance of formulating extensive differential diagnosis lists counters many of the cognitive biases in clinical practice such as availability bias by forcing the clinician to think of multiple alternative clinical hypotheses.

Other debiasing efforts include appropriate utilization of definitive diagnostic testing such MR imaging for neuroanatomical patient evaluation. The pragmatic reality of the spectrum of care scenarios, however, often limits the ability of the clinician to achieve a definitive diagnosis in all cases. This is a further impetus for the clinician to remain diligent in continual reevaluation, reassessment, and reaffirmation of any clinical diagnosis rendered under these conditions of uncertainty. In these instances, it is important to avoid becoming overconfident in what we believe is the patient’s disease especially when we are unable to definitively prove that disease process. A “healthy skepticism” should not limit our ability to continue managing patients but allows the clinician to be realistic as to what is known and equally as to what is unknown. There is a continual dynamic balance between “confidence” and “overconfidence” which allows the clinician to make medical decisions when actively caring for patients while maintaining “humility” in the knowledge that their assessments could be flawed.

Another option in maintaining receptiveness to collecting new information is to develop a mental reminder such “What else could be happening?”; “What more information is needed to confirm my assumptions?” or “What am I missing?”. These mantras may serve as prompts to be continually diligent in our clinical decision-making. It should also be considered that patients are unique entities with unique clinical manifestations of disease that may vary from stereotypical and historical descriptions. As a conscious effort to search for alternative explanations is important, deliberate practice in repeating these types of phraseology will help create dependable mental habits which support the continued search for the clinical truth.

Finally, teaching essential principles can support efforts in achieving an accurate clinical diagnosis in patients with suspected neurologic disease. These include:

- Clinical signs of nervous system disease reflect the location of the abnormality in the nervous system *not* the etiological cause.
- “Presumptive” diagnosis is the most likely clinical decision to be in error as these conclusions are not “definitive” diagnoses.
- The most extensive and inclusive differential diagnosis list is most likely to lead to the identification of the definitive diagnosis.
- Utilization of MR imaging is the most available ante mortem diagnostic modality to provide the diagnosis of anatomical neurologic diseases in the contemporary era of veterinary neurologic practice.

## Conclusion

Cognitive biases and resultant cognitive errors are pervasive within clinical neurologic practice but equally within all clinical decision-making regardless of which body system is abnormal. The initial step in attempting to minimize or eliminate the cognitive errors from the clinical evaluation process is becoming consciously aware of these biases and the associated operational situations where these cognitive errors are more likely to enter the medical decision-making process. Student education of these biases as they are developing their clinical skills is imperative. A case-based identification and debrief discussion can support learning of the influence of cognitive bias in neurological diagnosis. Finally, learning debiasing strategies to counterbalance the negative consequences of these thinking errors will improve clinical diagnosis and ultimately, patient outcomes.
